# XAS/DRIFTS/MS spectroscopy for time-resolved *operando* investigations at high temperature

**DOI:** 10.1107/S160057751801305X

**Published:** 2018-10-23

**Authors:** G. Agostini, D. Meira, M. Monte, H. Vitoux, A. Iglesias-Juez, M. Fernández-García, O. Mathon, F. Meunier, G. Berruyer, F. Perrin, S. Pasternak, T. Mairs, S. Pascarelli, B. Gorges

**Affiliations:** a ERSF – European Synchrotron Radiation Facility, 71 Avenue des Martyrs, 38000 Grenoble, France; b Instituto de Catalisis y Petroleoquimica (ICP-CSIC), Marie Curie 2, Cantoblanco, 28049 Madrid, Spain; c Univ Lyon, Université Claude Bernard Lyon 1, CNRS, IRCELYON, 2 Avenue Albert Einstein, 69626 Villeurbanne, France

**Keywords:** combination XAS/DRIFT/MS, *operando* measurements, high temperature, small-dead-volume cells

## Abstract

The combination of complementary techniques in the characterization of catalysts under working conditions is a very powerful tool for an accurate and in-depth comprehension of the system investigated. In particular, X-ray absorption spectroscopy (XAS) coupled with diffuse reflectance infrared Fourier transform spectroscopy **(**DRIFTS) and mass spectroscopy (MS) is a powerful combination since XAS characterizes the main elements of the catalytic system (selecting the absorption edge) and DRIFTS monitors surface adsorbates while MS enables product identification and quantification. In the present manuscript, a new reactor cell and an experimental setup optimized to perform time-resolved experiments on heterogeneous catalysts under working conditions are reported. A key feature of this setup is the possibility to work at high temperature and pressure, with a small cell dead volume. To demonstrate these capabilities, performance tests with and without X-rays are performed. The effective temperature at the sample surface, the speed to purge the gas volume inside the cell and catalytic activity have been evaluated to demonstrate the reliability and usefulness of the cell. The setup capability of combining XAS, DRIFTS and MS spectroscopies is demonstrated in a time-resolved experiment, following the reduction of NO by Rh nanoparticles supported on alumina.

## Abstract 2

A new reactor cell and experimental setup designed to perform time-resolved experiments on heterogeneous catalysts under working conditions that simulltaneously combines XAS, DRIFT and MS spectroscopies are reported.

## 1. Introduction   

The correlation of catalyst performance with its local and electronic configuration is a major challenge for the scientific community. In particular, the comprehension of interactions between reactants and catalysts, formation of reaction intermediates and recognition of active sites are of paramount importance in the catalyst design, improving efficiency, selectivity and lifetime. The study of catalysts under working conditions is essential for a complete comprehension of their structure–function relationship. Moreover, the combination of complementary characterization techniques is very powerful since it allows investigation from different perspectives in real time and correlation with the catalyst behaviour subject to the same conditions (*e.g.* temperature, gases and pressure).

X-ray absorption spectroscopy (XAS) is widely used for *in situ* and *operando* experiments since it can provide information on the local and electronic structures of the absorbing elements (Bordiga *et al.*, 2013 ▸). Since the first experiment performed by Couves *et al.* (1991 ▸), coupling XAS and X-ray powder diffraction (XRD) (Couves *et al.*, 1991 ▸), several cells and experimental setups were developed for *in situ* or *operando* investigation, combining XAS with a complementary technique (Clausen & Topsøe, 1991 ▸; Beale *et al.*, 2005 ▸; Frenkel *et al.*, 2011 ▸; Tinnemans *et al.*, 2006 ▸). Among the different couplings available for the study of solid–gas heterogeneous catalysts, XAS, diffuse reflectance infrared Fourier transform spectroscopy (DRIFTS) and mass spectrometry (MS) are a powerful combination because characterization of the metallic centre, identification of surface adsorbates and quantification of reaction products are performed at the same time (Newton & Beek, 2010 ▸).

Experimental setups devoted to this combination have already been developed and hereafter a general overview is reported. Pioneering work devoted to the study of molecules was carried out by Young (Young & Spicer, 1990 ▸; Young, 1996 ▸) and concerning catalysis by Newton and co-workers on the ID24 beamline at the European Synchrotron Radiation Facility (ESRF, Grenoble, France). The first experiments were performed using a custom-built DRIFTS cell (Newton *et al.*, 2004 ▸), modifying a design proposed by McDougall (Cavers *et al.*, 1999 ▸). The flat-top design minimized the cell dead volume, resulting in a fast gas-switching response. However, the final design affected the cell maximum temperature (400°C) and presented a bypass of the gas feed in the catalytic bed. As a further step, a commercial DRIFTS cell provided by Spectra-Tech was modified (Newton, 2009 ▸). Two carbon-glass windows for X-rays were added to the original dome, resulting in a higher dead volume and the catalytic-bed bypass was not completely solved as underlined by Meunier *et al.* (2007 ▸, 2008 ▸). A similar design was adopted by another setup which combined XAS and DRIFTS and was developed at Brook­haven National Laboratory and mounted on the X18 beamline (Marinkovic *et al.*, 2011 ▸). It was assembled from a Harrick cell using a DaVinci arm attached to a modified Praying Mantis DRIFTS accessory. The cell was mounted in the internal sample chamber of the infrared (IR) spectrometer and it could work up to 500°C at ambient pressure. The Harrick cell equipped with Praying Mantis optics was also modified at Argonne National Laboratory, USA. The setup was initially designed for X-ray pair distribution function measurements, after which it was also used for XAS measurements in transmission and fluorescence geometries (Beyer *et al.*, 2014 ▸; Yao *et al.*, 2014 ▸). A relatively simple cell was used by Bando *et al.* (2009 ▸, 2012 ▸), placing the sample in pellet form in the centre of a cross-like cell measuring both X-ray and IR in transmission configuration. The sample was heated to 530°C and could sustain up to 3 bar of pressure, but with a large dead volume and bed-bypass problem. Later, a cell able to combine XAS and DRIFTS with a different design was proposed by Chiarello *et al.* (2014 ▸). The novelty of this cell was that both X-rays and IR radiation passed through the same window in direct contact with the sample. In this way, a plug-flow reactor with reduced dead volume was achieved. Fast exchange of gases became possible, fulfilling the requirements for transient experiments. The main drawbacks of this design were the necessity to drill a small hole of 0.5 mm diameter in the CaF_2_ IR window for XAS spectra acquisition and to seal it using a carbon-based glue with high thermal stability. The cell was tested up to 500°C at ambient pressure. Moreover, the design of the cell allows the user to perform XAS measurements alone or combined with XRD and IR at the same time (Marchionni *et al.*, 2017 ▸). An innovative approach was recently proposed by Urakawa and co-workers, which combined XAS or XRD with IR spectroscopy on a pellet-shaped sample (Hinokuma *et al.*, 2018 ▸). The flexibility of a modular IR interferometer (Arcoptix SA, OEM model) allowed measurements in both transmission and diffuse-reflection modes by changing the relative position of the IR source and IR radiation detector. Notwithstanding the difficulties during sample preparation to fulfil the different sample requirements for IR and XAS or XRD techniques, investigation of samples in transmission configuration in pellet form often results in higher data quality.

The general concept followed in designing the catalytic reactor and presented in this manuscript was to develop a flexible cell able to perform *operando* measurements combining XAS and IR spectroscopies for solid–gas reaction catalysts. According to this idea, the design proposed by McDougall (Cavers *et al.*, 1999 ▸) was the most suitable to fulfil our requirements. Indeed, this configuration allows for the optimization of radiation windows for both IR and X-rays and hosts the sample in free powder form. Both points are essential to cover a wide range of experimental conditions (*e.g.* X-ray energy, metal loading, different supports) and perform *operando* experiments. Moreover, the experimental setup was optimized to minimize cell dead volume, avoid any sample bypass for gases and work at high temperatures under high pressure.

In the present manuscript, we describe the cell and the experimental setup developed to combine XAS, DRIFTS and MS. First, the cell was extensively investigated in the laboratory and the following tests were performed: sample surface temperature in comparison with cell body temperature, evaluation of gas-exchange time inside the reaction chamber and evaluation of reactor capabilities during CO hydrogenation over a Sn–Co/Al_2_O_3_ sample. The setup was then mounted on beamline ID24 at ESRF (Pascarelli *et al.*, 2016 ▸) to demonstrate the cell capability, combining XAS, DRIFTS and MS spectroscopies during a time-resolved experiment following the reduction of NO by Rh nanoparticles supported on Al_2_O_3_.

## 2. Experimental   

### 2.1. *Operando* XAS/DRIFTS/MS cell   

**Figure 1:**
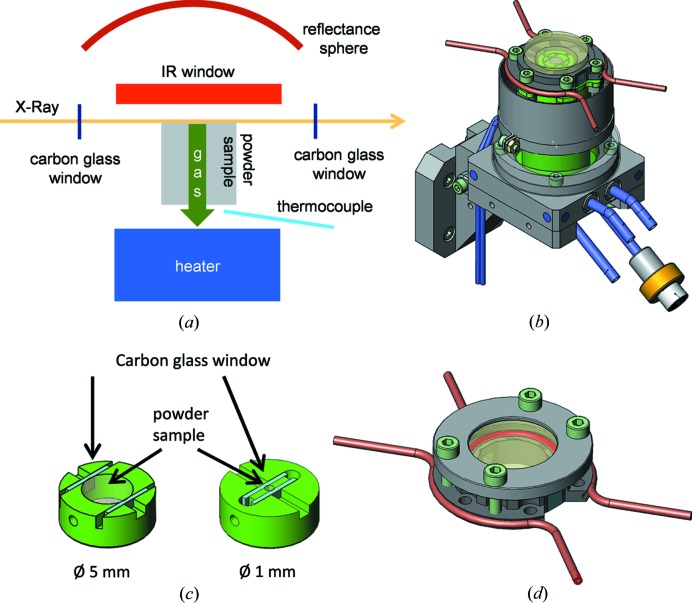
(*a*) Concept design of the cell. Illustrations of (*b*) the cell, (*c*) the sample holders and (*d*) the pressure dome.

The cell developed was designed to allow the simultaneous combination of XAS, DRIFTS and MS spectroscopies in order to investigate the sample under working conditions. This aim implies application of temperature, pressure and reactive atmosphere to the catalyst. In addition, to optimize solid–gas interaction, catalysts are measured in powder form without making a pellet, allowing reactive gases to flow through the catalytic bed. The concept design is schematized in Fig. 1 ▸(*a*), and Fig. 1 ▸(*b*) displays a sketch of the cell. XAS measurements are performed in transmission configuration while IR spectra are collected in diffuse reflection mode by the reflectance sphere (DRIFTS). The cell is composed of two parts: the main body and the dome. The body hosts the heater and thermocouple, sample holder and gas system, while the dome hosts IR and X-ray windows. The sealing between dome and body is guaranteed by a metal CF16 O-ring clamped by six screws. Cu metal is usually used but Au coating can be applied on its surface if necessary to avoid any chemical reaction. This configuration is very convenient because it makes the setup flexible for future developments; minor changes in the body and in the dome are sufficient to implement new experiments and cell capabilities (Castillejos-López *et al.*, 2017 ▸). The sample in free powder form is hosted in a crucible that can be easily changed, optimizing sample thickness for XAS measurements performed in transmission mode according to metal loading and absorption edge. The X-ray path can be tuned from 1 mm to 5 mm: the powder is placed between two carbon-glass windows that are transparent to X-rays, see Fig. 1 ▸(*c*). A metal grid below the powder allows the passage of gas through the catalytic bed. Particular attention was paid to the design and machining of the sample holder in order to avoid any bypass of gas without interaction with the sample. As illustrated in Figs. 1 ▸(*a*) and 1 ▸(*b*), a round window of 25 mm diameter and two square 5 mm × 5 mm windows are mounted for DRIFTS and XAS measurements, respectively. Both can be easily changed, tuning material and thickness according to experimental requirements. In the typical configuration, 2 mm-thick CaF_2_ and 200 µm-thick carbon-glass windows for IR and X-rays are used, respectively. Since minimization of dead volume is a mandatory requirement when performing fast kinetic studies, the design was optimized accordingly: the three windows are directly glued to the dome and the distance between the sample surface and the IR window is 1 mm. This design yields a reaction chamber dead volume of 0.5 cm^3^. This also guarantees a large solid angle for the diffuse reflectance sphere in order to also collect good signal-to-noise spectra during kinetic experiments. The silicon glue utilized (LOCTITE SI 5399) can sustain temperatures up to 350°C and, in addition to temperature and chemical stability, a fundamental feature of this glue is its elasticity. In fact, when heating the cell at high temperatures, the different thermal expansion of the glass window and metal flange can result in damage or breaking of the glass window; the role of the glue is to minimize the glass–metal mechanical strain to preserve the integrity of the window.

Operation temperatures range from room temperature (RT) to 600°C. The reactive feed can be pre-heated before interaction with the sample in order to minimize any thermal gradient along the catalytic bed. A *K*-type thermocouple is placed between the sample holder and heater, though outside the reaction chamber in order to avoid any interaction with the reactive atmosphere. The cell is made of Inconel alloy for its resistance to reducing and oxidizing atmospheres even at high temperatures. Both inlet and outlet pipes can be heated to 150°C to avoid liquid condensation.

It is important to note that the cell configuration implies the use of four carbon-glass windows (two in the sample holder hosting the powder and two in the dome) and their X-ray absorption has to be considered in the design of the experiment, particularly at low energy. The minimum window thickness successfully tested is 60 µm each, resulting in a total of 240 µm of carbon glass. Even using low-density material (ρ_carbonglass_ = 1.5 g cm^−3^) its contribution at low energy can be significant; for example, it results in a total absorption of μ*x* = 0.66 at the Ti *K*-edge (4966 eV).

The cell can be also equipped with a second dome in order to work up to 5 bar of pressure. In this case, the IR window is not glued but clamped by a metal flange while the two carbon-glass windows are glued in the inner part of the dome, see Fig. 1 ▸(*d*). This configuration implies an increase in the distance between the sample surface and IR window up to 3 mm and thus the dead volume reaches 1 cm^3^.

### 2.2. Experimental setup mounted on ID24   

**Figure 2:**
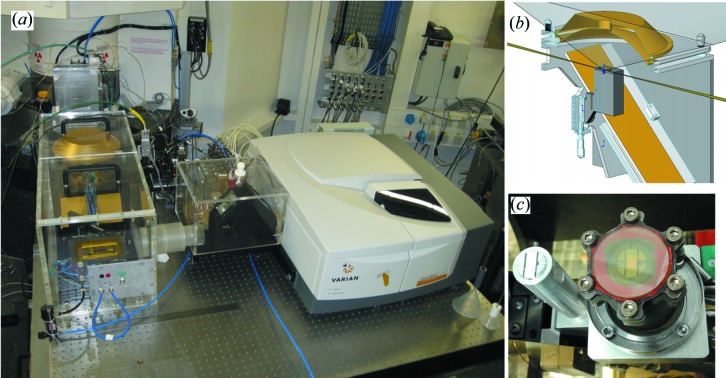
(*a*) XAS/DRIFTS/MS setup mounted on ID24. (*b*) Schematic of the DRIFTS cell mounted inside the reflectance sphere. (*c*) Photograph of the top of the cell.

The experimental setup combining XAS, DRIFTS and MS and hosting the cell described above was developed specifically to be used on beamline ID24 at the ESRF (Pascarelli *et al.*, 2016 ▸); however, since it is mounted on top of one plate, it can be hosted on any XAS beamline equipped for *operando* studies of catalysts (Castillejos-López *et al.*, 2017 ▸), and an overview is given in Fig. 2 ▸(*a*). The combined XAS–DRIFTS system is composed of a Fourier transform infrared (FTIR) commercial instrument Varian 680, a diffuse reflectance sphere provided by OMT Solutions, the cell described in the previous section and a setup to handle gases according to experimental requirements, *e.g.* mass-flow controllers, saturator and fast switching valves. The whole setup is mounted on a 1.2 m × 1.5 m table motorized along the three axes, allowing placement of the sample in the correct position with respect to the X-rays, keeping, at the same time, the IR optics and the spectrometer fixed. This solution guarantees the correct alignment of the IR optics with respect to the sample surface during the experiment. A set of Au-coated mirrors together with the diffuse reflectance sphere focus IR radiation to the surface of the sample. The backscattered light, reflected by the same mirrors, enters an MCT (mercury cadmium telluride) external detector after passing through a beam splitter. The sample surface must be placed in the focal point of the reflectance sphere and the alignment is achievable by a vertical movement of the cell. In addition, three fine motions can adjust the angle of the spherical mirror. The whole setup is mounted inside a Plexiglas box under pure N_2_ flux to decrease H_2_O and CO_2_ vibrational modes in the IR spectra. One critical issue combining IR and XAS spectroscopy is the different volume and portion of the catalytic bed investigated. On one hand, IR spectroscopy investigates only the top part of the catalytic bed since the typical penetration depth for IR light in solid matter is of the order of few tens of micrometres (Mondelli *et al.*, 2006 ▸). On the other hand, XAS measured in transmission mode relies on the absence of the incident-beam (*I*_0_) leaks present in the *I*_1_ beam, which forces us to place the X-ray beam just below the surface. In fact, the sample–air interface on the top part of the catalytic bed is never well defined since catalysts are hosted in the sample holder in free form and the sample grains move as a result of temperature and gas flow. The microbeam size available on ID24 and the geometry of the sample holder minimize this issue, although it was not possible to avoid it completely.

This experimental setup is optimized to perform kinetic studies on catalysts under working conditions. The general approach for these kinds of studies foresees to follow, with a suitable time resolution, the modifications occurring in the local and electronic structure of the catalyst, surface adsorbates of active sites and product formation, as the conditions change, *e.g.* from inert to reactive atmosphere or from dark to UV–Vis light. In the present manuscript, XAS experiments performed to validate the setup were carried out at the Energy Dispersive XAS beamline (ID24) at ESRF (Pascarelli *et al.*, 2016 ▸) because its sub-millisecond time resolution made this beamline particularly suitable for exploring cell performances. This capability requires precise synchronization between X-ray detector, IR spectrometer, mass spectrometer and the device used to change the catalytic conditions of the sample. Two general requirements need to be fulfilled: a capability to follow evolution of samples right after condition modification and the possibility to correlate, at any time, the spectra of the three techniques with the experimental conditions. For this aim, an OPIUM timing and synchronization card drives all devices. The frames collected using an X-ray detector are used as counter in the macro during data acquisition. At the beginning of each experiment the OPIUM starts the acquisition of the X-ray detector and of the IR spectrometer. It can trigger a change in state of other equipment. For example, it can open/close a shutter for UV–Vis in photochemistry experiments or open/close a gas switching valve for solid–gas reaction interactions. Any further change can happen only after a defined number of frames acquired by the X-ray detector. This approach guarantees a precise control of the experimental conditions, avoiding changes in the middle of acquisition of one spectrum and at the same time allowing the correlation of information extracted by XAS, IR and MS spectroscopies with the experimental conditions applied to the catalysts. Moreover, it is very flexible since different synchronization schemes can be implemented and several devices can be driven. The standard configuration is able to control, in addition to the X-ray detector and the IR spectrometer, up to three switching valves and another device at the same time and independently from each other. The mass spectrometer is not synchronized because it works in continuous mode; however, the OPIUM signal can be recorded by the MS software in order to monitor changes in the gas phase and relate them with the electronic/structure/surface changes.

During XAS measurements performed in the energy-dispersive configuration, the incident intensity *I*_0_ is measured either before or after the transmitted intensity *I*_1_ using the same detector. In catalysis, most of the time *I*_0_ is collected through the catalyst support. In this way, *I*_0_ normalization more efficiently eliminates effects caused by X-ray–sample interaction other than photo-absorption (such as small-angle scattering from the support). Considering this, a second sample holder, visible on the left of Fig. 2 ▸(*c*), was mounted to host the pure support of the catalysts.

### 2.3. Samples for laboratory and beamline measurements   

Two different experiments were carried out to evaluate the performance of the cell: CO hydrogenation over a Sn–Co/Al_2_O_3_ catalyst and NO–CO reaction over a Rh/Al_2_O_3_ catalyst.

The CO hydrogenation was performed without X-rays to evaluate catalytic performance and hence compare the results with other reactors. Details about the synthesis, characterization and catalytic evaluation of the Sn–Co/Al_2_O_3_ are reported elsewhere (Paredes-Nunez *et al.*, 2018 ▸). In brief, the cobalt loading was 14.4 wt% and that of Sn was 0.52 wt%, yielding a Sn/Co molar ratio of 1:60 with a metal dispersion of *ca* 9.2%. The Sn–Co/Al_2_O_3_ sample was reduced *in situ* in a stream of H_2_ before being exposed to a flow of syngas (H_2_:CO = 2) at 220°C. The cell effluent was analysed by 2 m path-length transmission IR gas cell (Paredes-Nunez *et al.*, 2015 ▸) that enabled determination of the concentration of methane, propene and methanol through calibration curves. The rates of formation (expressed in mol g_catalyst_^−1^s^−1^) were calculated and compared with those reported elsewhere (Paredes-Nunez *et al.*, 2018 ▸) obtained on a modified high-temperature low-pressure Spectra-Tech DRIFTS cell (Meunier *et al.*, 2008 ▸).

The NO–CO reaction over a Rh/Al_2_O_3_ catalyst was performed in order to validate the whole setup and the combination of spectroscopies (XAS+DRIFTS+MS). The sample was composed of 5 wt% Rh nanoparticles supported on γ-Al_2_O_3_ (Sigma-Aldrich, 212857). The Rh/Al_2_O_3_ catalyst was reduced *in situ* (5%H_2_/He, 250°C, 10°C min^−1^, 30 min), the temperature was increased to 275°C and reduction of NO by CO was performed. Two streams were alternated using a switching valve: first 5%NO/He and then 5%CO/He. Each stream was kept for 60 s. Coupled XAS, DRIFTS and MS measurements were performed. Spectra in transmission mode at the Rh *K*-edge (23220 eV) were collected using a Si(111) polychromator in a Laue configuration and a Hamamatsu detector (Kantor *et al.*, 2014 ▸). A Varian 680 FTIR instrument collected spectra in DRIFTS mode. Both measurements were performed with a time resolution of 50 ms per spectrum. The infrared background was considered as the first spectrum under CO to evaluate its evolution on the particle surface. The gas outlet was measured by a Hidden Analytical HPS-20 QIC MS (intensity measured for ten masses corresponding to different gases) with a time resolution of 300 ms. The XAS data reduction, both spectra normalization and extraction of EXAFS signals in the *k* range 3–11 Å^−1^, was performed in batch mode by the XAS plug-in from *PyMca* described elsewhere (Cotte *et al.*, 2016 ▸).

## 3. Results and discussion   

This section is divided in two different parts. In the first, the cell performance as a catalytic reactor is explored, investigating sample temperature, time for gas exchange and catalytic activity. The second part is focused on the cell and setup capabilities, combining XAS, DRIFTS and MS.

### 3.1. Laboratory performance investigations: temperature, dead volume and catalytic tests   

#### 3.1.1. Pyrometry test   

**Figure 3:**
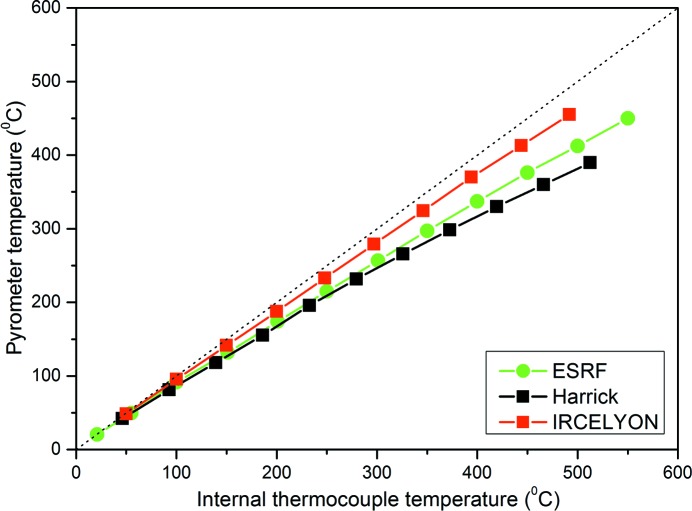
Internally *versus* externally measured temperature for the indicated cells.

Temperature control of the cell has been evaluated using an optical pyrometer. The temperature given by the thermocouple internally fixed to the cell body was compared with that determined from the thermal radiation emitted by the surface of the sample. The results of the tests on the present cell are compared with those on the Harrick and Spectra-Tech (IRCELYON) model (Li *et al.*, 2013 ▸) cells in Fig. 3 ▸. As the thermocouple is placed below the sample holder, the temperature measured by the thermocouple is actually a poor estimate of the sample surface temperature. Yet, the deviation is constant at a given temperature (*i.e.* independent of gas composition and flow rate) and, once known, the set point can be adjusted to make the cell reach the appropriate temperature during experiments.

#### 3.1.2. Dead volume test   

**Figure 4:**
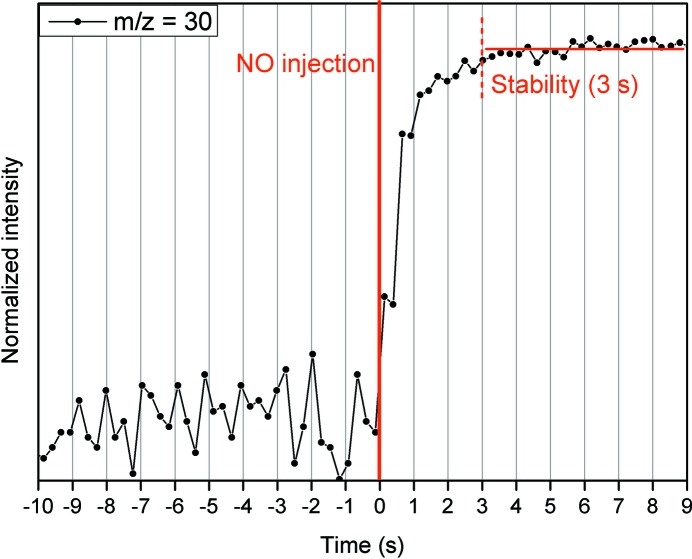
Time evolution of the MS signal corresponding to *m*/*z* = 30 before and after the injection of NO.

An indication of the dead volume of the cell is provided by the time required for a known flow of gases to replace the previous atmosphere. A way of evaluating this time consists of calculating the time between the gas-phase switch and the arrival at the detector of this new gas phase. Several tests were performed and one example is shown in Fig. 4 ▸: at time = 0 s, the neutral atmosphere flowing through the cell is exchanged by a NO-containing one. Only 3 s are required using 75 ml min^−1^ for the signal corresponding to NO (**m**/*z* = 30) to reach stability (within the error).

#### 3.1.3. Catalytic test: CO hydrogenation   

The catalytic performance of the cell was assessed and compared with that of a modified Spectra-Tech model (Meunier *et al.*, 2008 ▸) from the IRCELYON laboratory for CO hydrogenation (*i.e.* Fischer–Tropsch synthesis) at atmospheric pressure (Paredes-Nunez *et al.*, 2018 ▸). It must be stressed that the modified Spectra-Tech cell was shown to yield identical reaction rates as those measured in a traditional plug-flow reactor (Meunier, 2010 ▸), at least for temperatures below 300°C.

**Figure 5:**
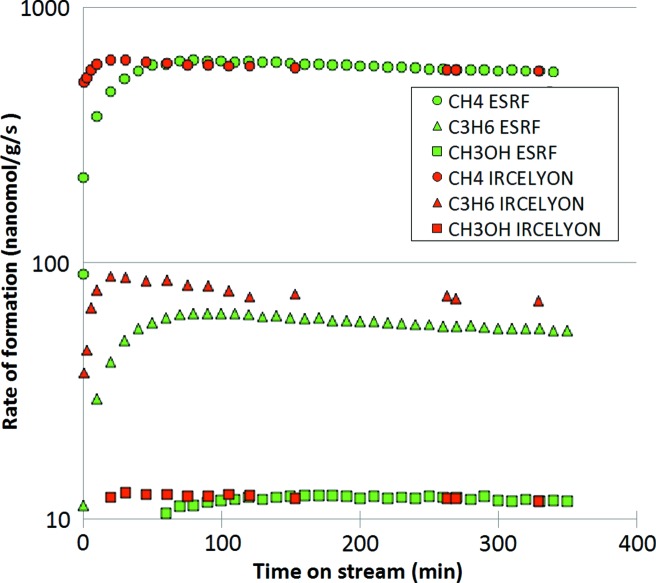
Comparison of the catalytic activity for CO hydrogenation (H_2_/CO = 2, 220°C, atmospheric pressure) in terms of reaction rates measured with the present cell and a modified Spectra-Tech cell (Paredes-Nunez *et al.*, 2018 ▸).

When flowing the syngas mixture at 220°C, the main products were methane, propene and methanol. Fig. 5 ▸ reports the reaction rates obtained for these main products over the first 6 h on stream. The rates measured were essentially identical for both cells, apart from the initial stream, which may have been caused by differences in lines and cell dead-volumes, thermal stabilization (owing to differences in the cell heat capacity) or transient contamination by air leading to a temporary deactivation of the metallic cobalt catalyst. These data show that the catalytic data obtained at steady-state using the present ESRF DRIFTS cell are fully consistent with those typically obtained on calibrated cells for a reaction that is very sensitive to O_2_ and moisture impurities.

### 3.2. Combined XAS/DRIFTS/MS test on ID24 beamline at ESRF: CO oxidation by NO   

CO oxidation by NO on 5% Rh nanoparticles was performed in order to test our experimental setup.

**Figure 6:**
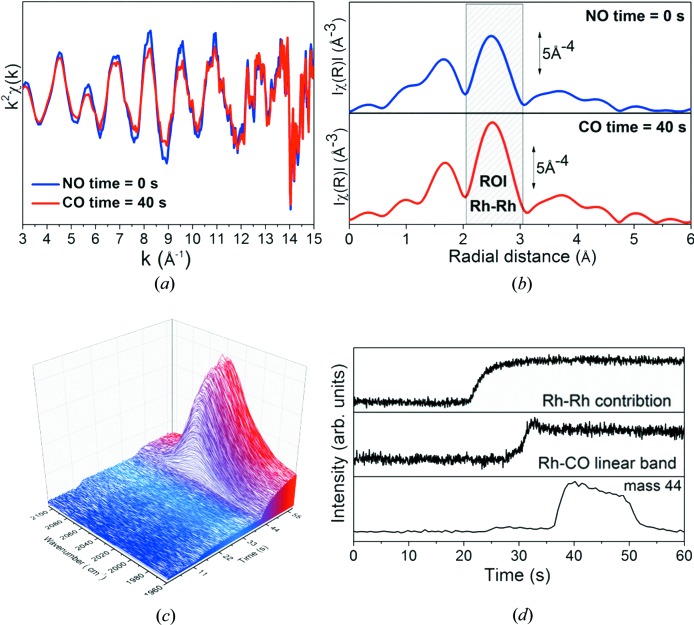
(*a*) *k* ^2^χ(*k*) XAS spectra collected on a Rh catalyst under CO (red curve) and NO (blue curve). (*b*) Corresponding |FT| spectra, *k* ^2^-weight not phase corrected, reported in part (*a*). (*c*) Evolution of DRIFTS spectra in the 1950–2100 cm^−1^ region. (*d*) ROI analysis performed by *PyMca* on EXAFS, DRIFTS and MS data.

At 275°C, the gas feed was changed from NO to CO and XAS, DRIFTS and MS data were recorded. Fig. 6 ▸(*a*) shows two selected EXAFS spectra in *k* space under a different atmosphere (blue curve under NO, red under CO), demonstrating the good data quality up to 11 Å^−1^ collected in 50 ms (average of the accumulation of 50 frames of 1 ms). The corresponding *k* ^2^-weighted not phase-corrected Fourier transform moduli (|FT|) are reported in Fig. 6 ▸(*b*). Both spectra show two different contributions corresponding to Rh−O and Rh−Rh paths at 1.6 Å and 2.5 Å, respectively. Evolution of DRIFTS data in the 1950–2100 cm^−1^ region is reported in Fig. 6 ▸(*c*). Initially no bands were observed but within 28 s an increasing contribution centred at 2025 cm^−1^ and ascribed to a linearly adsorbed CO band (Yang & Garl, 1957 ▸) appeared. The evolution of the observed feature was followed by the region of interest (ROI) option implemented in *PyMca* and described elsewhere (Cotte *et al.*, 2016 ▸). The time evolution of the |FT| peak area of Rh–Rh, the CO band area and the 44 mass signal obtained from EXAFS, DRIFTS and MS characterization techniques, respectively, are shown in Fig. 6 ▸(*d*).

Switching the gas phase from NO to CO reduced the rhodium particles, as indicated by the shift in the *K*-edge towards lower energies (not shown) and the decrease in the contribution from the first O shell in the EXAFS spectra. In addition, the growing intensity of the second shell (corresponding to Rh neighbours) points to the agglomeration of the particles. Only after the stabilization of the EXAFS spectra does the infrared band ascribed to CO adsorbed over Rh^0^ increase. From the MS results, an increase in the mass 44 signal, attributed to CO_2_ or N_2_O, was observed very faintly above 25 s and more significantly after 36 s. This combined information indicates that the CO molecules replace the NO molecules adsorbed over the Rh particles, initially reducing surface Rh atoms (0–22 s). Then, the CO induces agglomeration of Rh particles which leads to a general reduction of Rh atoms (20–30 s) and the materializing of Rh^0^–CO species (27–60 s). Oxidation of CO leads to some CO_2_ production at first (25–35 s), but most of the generation happens once the Rh particles are totally reduced (35–55 s). More information about this experiment and deeper analysis of the results can be found elsewhere (Monte *et al.*, 2018 ▸).

## 4. Conclusions   

A catalytic reactor for XAS/DRIFTS/MS combination was developed and successfully tested with and without X-rays present. The experimental setup was developed to perform time-resolved experiments on heterogeneous catalysts under working conditions. The cell design was optimized to obtain a low dead volume for the reaction chamber (around 0.5 cm^3^), allowing at the same time measurements up to 600°C. Measurements under pressure are possible up to 5 bar with an appropriate dome but at the expense of a larger dead volume (1 cm^3^). The design of the cell allows future developments, including different dome geometries with minor modifications of the main cell body. In this way, the heating part and the gas pipe system, critical components for catalytic application, remain unchanged. The combination and correlation of information from XAS, DRIFTS and MS spectroscopies are guaranteed by the synchronization of the X-ray detector, IR spectrometer, mass spectrometer, switching valve and other devices such as the UV–Vis shutter. Catalytic tests performed with and without X-rays confirmed the reliability and accuracy of the kinetic data obtained in the cell. Similar geometric cell configurations were previously reported, yet our design enables a low dead volume, accurate catalytic performance, high temperature and utilization at a few bars of pressure. The time-resolved capability of the setup was demonstrated by following the evolution of the Rh–Rh EXAFS contribution, the infrared band associated with Rh^0^–CO (2025 cm^−1^) and the signal of *m*/*z* = 44 corresponding to generated CO_2_.
